# Food-drug interactions knowledge and clinical competence among registered dietitians

**DOI:** 10.1186/s12909-026-09509-9

**Published:** 2026-06-06

**Authors:** Sofia Beirão, Carina Rossoni, Cíntia Ferreira-Pêgo, João Guilherme Costa

**Affiliations:** 1 School of Health Sciences and Technologies, Universidade Lusófona, Lisbon, Portugal; 2 Institute of Environmental Health (ISAMB) at the Faculty of Medicine of the University of Lisbon, Lisbon, Portugal; 3 Universidade Lusófona’s Research Center for Biosciences and Health Technologies (CBIOS), Lisbon, Portugal

**Keywords:** Food-drug interactions, Dietitians, Nutritionists, Clinical nutrition, Healthcare education, Patient safety

## Abstract

**Background:**

Food-drug interactions (FDIs) are increasingly prevalent due to chronic disease and polypharmacy and may compromise therapeutic efficacy and patient safety. Registered dietitians play a critical role in identifying and preventing FDIs through dietary assessment and counselling in clinical practice; however, their knowledge and competence in this domain remain poorly characterised.

**Methods:**

A cross-sectional observational study was conducted using a structured self-administered online questionnaire among 151 registered dietitians, active members of the Portuguese Order of Nutritionists (*Ordem dos Nutricionistas*), in 2024. Knowledge of general and clinically relevant FDIs, prior education and training exposure, perceived adequacy of FDI knowledge, and self-reported training needs were assessed. Descriptive and comparative analyses examined knowledge patterns by professional experience and area of practice.

**Results:**

Participants demonstrated a good understanding of basic FDI concepts. However, specific knowledge of clinically relevant interactions averaged 58.72% (Sufficient), with markedly lower accuracy for interactions involving propranolol (22.5%) and warfarin (42.4–62.3%). Although most respondents (93.4%) reported prior academic exposure to FDIs, only 23.2% considered their knowledge sufficient for practice. Furthermore, 21.2% had undertaken complementary training beyond their bachelor’s degree, suggesting a felt need to supplement formal education. Greater knowledge was observed among registered dietitians working in clinical practice and those with more professional experience.

**Conclusions:**

Registered dietitians demonstrated foundational awareness of FDIs, but also important gaps in clinically relevant interaction knowledge and perceived competence. These findings highlight the need to strengthen FDI content in nutrition curricula and continuing professional development to support safe and effective practice in populations exposed to polypharmacy.

## Introduction

We are currently living in an era where chronic diseases are becoming increasingly prevalent and represent a major public health challenge on a global scale. This problem is rapidly escalating due to population growth and the ageing of societies [1].

In Portugal, approximately 57.8% of the population aged 25–74 years suffers from at least one chronic disease, equating to around 3.9 million people [2]. This percentage is higher than the European average, where approximately 36% of the population is affected by chronic diseases, corresponding to more than 150 million people [3].

As a result, the number of polymedicated individuals has been increasing [4], with different pathologies such as cancer and cardiovascular diseases [2]. In the chronic treatment of various pathologies, patients take multiple drugs daily, which exposes them to a greater risk of not only pharmacological interactions but also food-drug interactions (FDIs) [5]. Likewise, elderly patients with chronic diseases such as diabetes and hypertension, who take three or more medications per day, are particularly susceptible to FDIs [6]. In a study by Onder et al. that examined the prevalence of polymedicated individuals in 57 nursing homes across 8 European countries, a sample of 4,023 users with an average age of 83.5 years was analysed [7]. The study found that 49.7% were polymedicated, while 24.3% were excessively polymedicated, taking 10 or more different medications daily [7].

Polypharmacy thus emerges as a highly relevant global challenge, as it can lead to adverse effects that may compromise the safety and efficacy of therapeutic interventions [8]. Bushra et al. argue that, in hospitalised patients, FDIs can trigger unnecessary adverse reactions, with the main consequence being an increase in morbidity or length of hospital stay [9]. According to recent data, it is observed that annually, 8.6 million individuals in Europe are admitted to emergency hospital services due to adverse drug effects. Of these admissions, it is estimated that 50% could have been avoided, as 70% involve patients over 65 years old who take five or more drugs daily [10].

Healthcare professionals in the medical and pharmaceutical fields have been expressing deep concern about this issue [10], which has led to the emergence of various approaches and guidelines to reduce polypharmacy and, consequently, protect each individual’s health [11–13].

Given pharmacokinetic alterations, nutrients can modify the effects of a drug by interfering with its absorption, distribution, metabolism, and excretion [14–16]. Regarding pharmacodynamic modifications, there may be changes in its mechanism of action or therapeutic target, which can result in decreased or increased drug efficacy, potentially leading to situations of inefficacy or toxicity [17]. Most pharmacokinetic interactions occur when foods alter the activity of enzymes and/or transporters involved in drug pharmacokinetics [16].

Since 1995, it has been reported that all healthcare professionals play an important role in identifying FDIs [18–20] and that the Joint Commission on Accreditation of Hospitals encourages healthcare professionals, particularly pharmacists and registered dietitians, to monitor FDIs in hospitalised patients and to provide guidance on this subject upon hospital discharge [21–24].

Thus, given the fundamental role registered dietitians play in creating dietary plans, they must possess a comprehensive understanding of FDIs. This expertise will enable them to optimise their patients’ therapeutic processes without compromising their safety or treatment [18]. In dietetics practice, the ability to identify and manage FDIs is central to medication-nutrition safety, particularly in populations exposed to polypharmacy. However, scientific evidence indicates significant gaps in knowledge on this topic among various healthcare professionals [25–30]. Therefore, given these knowledge gaps, it is important to analyse the curriculum structure of health professionals’ degrees, particularly for nutrition sciences, to assess whether there is sufficient training in FDIs and if the training provided is adequate to ensure safe clinical practice.

Despite the recognised role of registered dietitians in medication-nutrition care, evidence specifically examining their knowledge and competence in FDIs remains limited. A previous study with nutrition students identified foundational awareness but limited depth in clinically relevant FDI knowledge [31]; however, if these gaps persist into professional practice, it remains poorly characterised.

The purpose of this study was therefore to assess knowledge and perceived competence among registered dietitians, characterise their academic training, and evaluate perceived training needs relevant to their professional practice.

## Methods

### Study design and participants

The present observational, descriptive, cross-sectional study used a self-reported online questionnaire developed via Google Forms^®^. The study population comprised registered dietitians who were active members of the *Ordem dos Nutricionistas* in 2024, recruited through convenience sampling. At the time of data collection (May–June 2024), the *Ordem dos Nutricionistas* had approximately 5264 active members. As the questionnaire was disseminated via professional and social media networks rather than through direct individual invitation, the precise number of dietitians who viewed the invitation could not be retrieved, and a formal response rate cannot be calculated.

### Data collection and measures

Data were collected between May 7 and June 18, 2024. The questionnaire was disseminated through professional and personal networks, including social media platforms.

Participation was voluntary and anonymous. Access to the questionnaire was granted only after electronic informed consent was provided and eligibility as a registered dietitian was confirmed.

The questionnaires consisted of five sections. The first obtained informed consent. The second section collected sociodemographic characteristics (age, sex, nationality, and region of residence). The third section assessed the academic and professional background, including the highest degree, institution, year of graduation, years of experience, and area of nutrition practice. This section also evaluated exposure to formal education on FDIs, estimated instructional hours on this topic, perceived adequacy of training, need for further education, and the perceived importance attributed to FDI knowledge in clinical nutrition practice. The fourth and fifth sections of the questionnaire were based on a previously published instrument [32] and were adapted to the Portuguese context, including commonly prescribed medications. The fourth section assessed general knowledge of FDIs. The fifth section evaluated knowledge of specific clinically relevant FDIs and was subdivided into three parts, including specific examples of FDIs, items on drug-alcohol interactions, and self-reflection regarding the acquisition and dissemination of FDI knowledge. The questionnaire was not exclusively closed-format. Several multiple-choice items included an ‘Other, please specify’ option, which activated a free-text field in Google Forms^®^ when selected; completion of this field was optional. Additionally, Section 5 included two open-ended questions inviting participants to name the most frequent and most severe FDIs encountered in clinical practice. Responses to open-ended items were analysed descriptively, with qualitative categorisation and reporting of the most frequently cited interactions. Responses to ‘Other, please specify’ fields were reviewed but were too infrequent or insufficiently specific to be coded separately and were not included in the quantitative analysis.

The adaptation process involved contextualisation of the original items to the Portuguese clinical setting, including replacement of medication examples with drugs commonly prescribed in Portugal, followed by content review by a panel of three experts in clinical nutrition and pharmacology, who assessed item relevance and clarity. Minor wording adjustments were made based on expert feedback. A formal pilot study was performed in 10 individuals in the presence of a researcher to assess comprehension and face validity of the questionnaire.

The estimated completion time was approximately 15 min. All responses were treated confidentially and used exclusively for scientific purposes. This study was conducted in accordance with the Declaration of Helsinki and with Good Clinical Practice guidelines. Ethical approval was granted by the Ethics Committee of the School of Health Sciences and Technologies, Universidade Lusófona (approval P09-24).

### Statistical analysis

Descriptive analyses were performed to summarise questionnaire responses. Continuous variables are presented as means and standard deviations, and categorical variables as frequencies (*n*) and percentages (%). The primary analysis focused on the distribution of correct responses to the FDI knowledge items. The questionnaire was configured to require responses for all closed-format items before submission, resulting in complete data for these items across all 151 participants; no missing data management was required. Open-ended items were optional, and non-responses were recorded and reported descriptively. Following data export, all responses were reviewed for internal consistency, and no implausible values were identified. Given the binary nature of the knowledge items, formal outlier detection did not apply to the primary outcome variables.

Professional experience was dichotomised into ≤ 5 years and ≥ 6 years based on conventions in health professions research distinguishing early-career practitioners; years of experience were collected as a continuous variable and categorised by the research team at the analysis stage. The parametric Student t-test for independent samples for continuous variables was used, and the Pearson Chi-square (χ^2^) test for categorical variables. Statistical significance was set at *p* < 0.05.

All analyses were performed using Jamovi^®^ statistical software (version 2.4.11; Jamovi Project, Australia).

## Results

Among the 151 included participants, 95.36% (*n* = 144) were female, and 4.64% (*n* = 7) were male. Additionally, 62 participants had five or fewer years of professional experience, and 89 had six or more years. Registered dietitians with six or more years of experience were significantly older than those with five or fewer years of experience (36.04 ± 6.78 vs. 28.01 ± 5.04 years; *p* < 0.001). Regarding the area of practice, 77.48% (*n* = 117) worked primarily in clinical nutrition practice, whereas 22.52% (*n* = 34) worked in non-clinical nutrition fields, including collective food service and catering, community and public health nutrition, nutrition research and teaching, and food technology, innovation, and marketing.

The mean age of participants was 32.74 ± 7.28 years, and nearly all (99.34%, *n* = 150) were Portuguese. Participants were geographically distributed across Portugal, with the largest proportions residing in the Lisbon Metropolitan Area (28.48%, *n* = 43), followed by North (26.49%, *n* = 40), and Central (18.54%, *n* = 28) regions (Table 1).

**Table 1:** Demographic and professional characteristics of the study sample by years of experience and area of practice

	Total Population (*n* = 151)	Years of Experience		*p*-valueª	Area of Practice		*p*-valueª
		≤ 5 years (*n* = 62)	≥ 6 years (*n* = 89)		Clinical (*n* = 117)	Non-clinical (*n* = 34)	
Sex, % (*n*)							
Female	95.36 (144)	40.28 (58)	59.72 (86)	0.376	77.08 (111)	22.92 (33)	0.593
Male	4.64 (7)	57.14 (4)	42.86 (3)		85.71 (6)	14.29 (1)	
Age, years	32.74 ± 7.28	28.01 ± 5.04	36.04 ± 6.78	**< 0.001**	32.73 ± 7.47	32.82 ± 6.70	0.946
Nationality, % (*n*)							
Spanish	0.66 (1)	0.00 (0)	100.00 (1)	0.402	100.00 (1)	0.00 (0)	0.589
Portuguese	99.34 (150)	41.33 (62)	58.67 (88)		77.33 (116)	22.67 (34)	
Area of Residence, % (*n*)							
North	26.49 (40)	40.00 (16)	60.00 (24)	0.064	72.50 (29)	27.50 (11)	0.557
Center	18.54 (28)	53.57 (15)	46.43 (13)		78.57 (22)	21.43 (6)	
Lisbon Metropolitan Area	28.48 (43)	37.21 (16)	62.79 (27)		79.07 (34)	20.93 (9)	
West and Tagus Valley	7.28 (11)	45.45 (5)	54.55 (6)		81.82 (9)	18.18 (2)	
Setúbal Peninsula	5.30 (8)	12.50 (1)	87.50 (7)		87.50 (7)	12.50 (1)	
Alentejo	5.96 (9)	22.22 (2)	77.78 (7)		88.89 (8)	11.11 (1)	
Algarve	1.99 (3)	0.00 (0)	100.00 (3)		100.00 (3)	0.00 (0)	
Azores Autonomous Region	3.31 (5)	60.00 (3)	40.00 (2)		40.00 (2)	60.00 (3)	
Madeira Autonomous Region	2.65 (4)	100.00 (4)	0.00 (0)		75.00 (3)	25.00 (1)	

Data expressed as mean ± standard deviation or percentage (*n*) for continuous and categorical variables, respectively

Bold value indicates statistically significant difference (*p* < 0.05)

^a^*p*-values for comparisons between groups were obtained using Student’s t-test or Pearson χ² test, as appropriate

Regarding the participants’ educational background (Table 2), most registered dietitians held a bachelor’s degree (67.55%, *n* = 102), whereas 29.80% (*n* = 45) had completed a master’s degree, of whom the majority (84.44%, *n* = 38) specialised in the clinical area. When analysing the institutions where registered dietitians completed their bachelor’s degree, the most frequently reported institutions were the Faculty of Nutrition and Food Sciences, University of Porto (23.84%, *n* = 36), the Egas Moniz School of Health and Science (18.54%, *n* = 28), and the Lisbon School of Health Sciences (11.92%, *n* = 18). It was also observed that registered dietitians who graduated from institutions such as Polytechnic and University Higher Education Cooperative (CESPU), Jean Piaget Higher School of Health, and Atlântica University were exclusively represented in the group with six or more years of experience. In contrast, those who graduated from the Faculty of Medicine of Lisbon or the University of Trás-os-Montes and Alto Douro were represented only in the group with five or fewer years of experience, reflecting differences in institutional history and programme availability over time. Additionally, the mean time since bachelor’s degree completion was 9.19 ± 6.01 years.

**Table 2:** Educational attainment, training contact, and information sources related to food-drug interactions among registered dietitians

	Total Population (*n* = 151)	Years of Experience		*p*-valueª	Area of Practice		*p*-valueª
		≤ 5 years (*n* = 62)	≥ 6 years (*n* = 89)		Clinical (*n* = 117)	Non-Clinical (*n* = 34)	
Academic degree completed, % (*n*)							
Bachelor’s	67.55 (102)	46.08 (47)	53.92 (55)	**0.044**	74.51 (76)	25.49 (26)	0.360
Post-graduation	1.32 (2)	100.00 (2)	0.00 (0)		50.00 (1)	50.00 (1)	
Master’s	29.80 (45)	28.89 (13)	71.11 (32)		84.44 (38)	15.56 (7)	
Doctorate	1.32 (2)	0.00 (0)	100.00 (2)		100.00 (2)	0.00 (0)	
Institution where bachelor’s was completed, % (*n*)							
Lisbon School of Health Sciences	11.92 (18)	33.33 (6)	66.67 (12)	**0.044**	83.33 (15)	16.67 (3)	0.557
Coimbra School of Health Technology	9.93 (15)	60.00 (9)	40.00 (6)		80.00 (12)	20.00 (3)	
Faculty of Nutrition and Food Sciences, University of Porto	23.84 (36)	38.89 (14)	61.11 (22)		66.67 (24)	33.33 (12)	
Faculty of Medicine, University of Lisbon	1.99 (3)	100.00 (3)	0.00 (0)		66.67 (2)	33.33 (1)	
Atlântica University Institute	1.99 (3)	0.00 (0)	100.00 (3)		100.00 (3)	0.00 (0)	
Egas Moniz School of Health and Science	18.54 (28)	32.14(9)	67.86 (19)		85.71 (24)	14.29 (4)	
Polytechnic Institute of Bragança	3.97 (6)	33.33 (2)	66.67 (4)		66.67 (4)	33.33 (2)	
Polytechnic Institute of Leiria	7.28 (11)	45.45 (5)	54.55 (6)		72.72 (8)	27.27 (3)	
Catholic University of Porto	3.97 (6)	50.00 (3)	50.00 (3)		83.33 (5)	16.67 (1)	
University of Trás-os-Montes and Alto Douro	0.66 (1)	100.00 (1)	0.00 (0)		100.00 (1)	0.00 (0)	
University of Algarve	5.30 (8)	25.00 (2)	75.00 (6)		50.00 (4)	50.00 (4)	
Fernando Pessoa University	1.32 (2)	50.00 (1)	50.00 (1)		100.00 (2)	0.00 (0)	
Lusófona University	5.30 (8)	87.50 (7)	12.50 (1)		100.00 (8)	0.00 (0)	
Polytechnic and University Higher Education Cooperative (CESPU)	3.31 (5)	0.00 (0)	100.00 (5)		80.00 (4)	20.00 (1)	
Jean Piaget Higher School of Health	0.66 (1)	0.00 (0)	100.00 (1)		100.00 (1)	0.00 (0)	
Years since bachelor’s degree completion, % (*n*)	9.19 ± 6.01	4.63 ± 2.98	12.67 ± 5.38	**< 0.001**	9.00 ± 5.61	9.96 ± 7.45	0.488
Current area of nutrition practice, % (n)							
Clinical Nutrition	77.48 (117)	39.32 (46)	60.68 (71)	0.419	100.00 (117)	0.00 (0)	**< 0.001**
Non-clinical practice	22.52 (34)	47.06 (16)	52.94 (18)		0.00 (0)	100.00 (34)	
FDIs covered during academic training, % (*n*)							
Yes	93.38 (141)	43.97 (62)	56.03 (79)	**0.006**	79.43 (112)	20.57 (29)	**0.031**
No	6.62 (10)	0.00 (0)	100.00 (10)		50.00 (5)	50.00 (5)	
Subjects dedicated to the FDIs topic, % (*n*)							
Part of the program of 1 course	67.14 (94)	43.62 (41)	56.38 (53)	0.956	77.66 (73)	22.34 (21)	0.774
Part of the program of 2 or more courses	19.29 (27)	44.44 (12)	55.56 (15)		81.48 (22)	18.52 (5)	
Exclusive course on the topic	13.57 (19)	47.37 (9)	52.63 (10)		84.21 (16)	15.79 (3)	
Class hours dedicated to the FDI topic, % (*n*)							
1–2 h/ semester	30.00 (42)	52.38 (22)	47.62 (20)	0.615	76.19 (32)	23.81 (10)	0.175
1–2 h/ month	22.14 (31)	38.71 (12)	61.29 (19)		67.74 (21)	32.26 (10)	
1–2 h/ week	38.57 (54)	40.74 (22)	59.26 (32)		87.04 (47)	12.96 (7)	
> 2 h/ week	9.29 (13)	46.15 (6)	53.85 (7)		84.62 (11)	15.38 (2)	
Knowledge acquired during bachelor’s degree considered sufficient, % (*n*)	23.18 (35)	57.14 (20)	42.86 (15)	**0.027**	77.14 (27)	22.86 (8)	0.956
Undertook complementary FDI training, % (*n*)	21.19 (32)	12.50 (4)	87.50 (28)	**< 0.001**	75.00 (24)	25.00 (8)	0.705
During bachelor’s degree	6.25 (2)	0.00 (0)	100.00 (2)	0.581	0.00 (0)	100.00 (2)	**0.011**
After bachelor’s degree	93.75 (30)	13.33 (4)	87.67 (26)		80.00 (24)	20.00 (6)	
Sources of information used to search for FDIs, % (*n*)							
Drug package leaflets	18.54 (28)	28.57 (8)	71.43 (20)	0.137	82.14 (23)	17.86 (5)	0.513
Specialised books on FDIs	7.28 (11)	18.18 (2)	81.82 (9)	0.109	54.55 (6)	45.45 (5)	0.059
Scientific articles	30.46 (46)	47.83 (22)	52.17 (24)	0.263	80.43 (37)	19.57 (9)	0.565
Institutional websites (e.g., Infarmed, research centres, or universities)	40.40 (61)	39.34 (24)	60.66 (37)	0.724	73.77 (45)	26.23 (16)	0.369
Other healthcare professionals	5.96 (9)	33.33 (3)	66.67 (6)	0.627	100.00 (9)	0.00 (0)	0.095
Other websites	1.32 (2)	100.00 (2)	0.00 (0)	0.088	100.00 (2)	0.00 (0)	0.443
Importance attributed to FDIs in clinical nutrition practice, points ^*^	4.70 ± 0.61	4.63 ± 0.68	4.74 ± 0.55	0.267	4.67 ± 0.64	4.76 ± 0.50	0.454

Data expressed as mean ± standard deviation or percentage (*n*), for continuous or categorical variables, respectively

*Abbreviations*: *FDIs* Food-drug interactions

Bold values indicate statistically significant differences (*p *< 0.05)

^a^ p-values for comparisons between groups were obtained using Student’s t-test or Pearson χ² test, as appropriate

^*^ Score ranged: 0–5

Among the 151 registered dietitians surveyed, 93.38% (*n* = 141) indicated that the topic of FDIs was covered during their academic training, whereas 10 individuals (6.62%) reported that it was not. All respondents who reported the absence of FDIs training had six or more years of professional experience, indicating a significant relation between years of experience as a registered dietitian and FDIs curricular coverage during the bachelor’s degree (*p* = 0.006).

Among registered dietitians who reported contact with FDI education, the topic was most commonly included in a single course (67.14%, *n* = 94), with no predominant pattern in instructional hours. Nevertheless, most participants (76.82%, *n* = 116) reported needing additional training on FDIs during their bachelor’s degree. Additionally, 21.19% (*n* = 32) pursued complementary training on FDIs, primarily after graduation (93.75%, *n* = 30), to improve knowledge and professional practice. Among those registered dietitians who pursued additional training, this included postgraduate programmes in nutrition and supplementation, certification in functional nutrition, master’s degrees in clinical nutrition, and workshops or courses provided by academic associations and institutions, or employers (data not shown).

Participants reported primarily searching for FDI information on institutional websites such as the Infarmed - Portuguese Authority of Medicines and Health Products, research centres, and universities (*n* = 61), and in scientific articles (*n* = 46). No statistically significant differences were observed by years of experience or practice area. However, all respondents who reported consulting other health professionals for FDI information (5.96%, *n* = 9) were from the clinical practice group and had six or more years of experience (66.67%, *n* = 6).

Regarding the importance of FDI knowledge in clinical practice, participants reported a high mean score of 4.70 ± 0.61 (scale 0–5), with no significant differences across experience or practice groups.

As detailed in Table 3, 99.34% (*n* = 150) of the sample correctly acknowledged that food and beverages can interfere with the therapeutic effect of a drug. However, a slightly lower proportion (91.39%, *n* = 138) recognised that such interference may increase toxicity or decrease drug efficacy, with no statistically significant differences across experience or practice groups. Participants also identified several factors influencing FDIs, including polypharmacy (84.77%, *n* = 128), drug dosage/regimen (82.12%, *n* = 124), diseases such as liver or kidney failure (74.17%, *n* = 112), and non-oral type of diet (enteral/parenteral) (61.59%, *n* = 93). Significant statistical differences by years of experience were observed for the recognition of genetic factors (*p* = 0.024) and polypharmacy (*p* = 0.041), with registered dietitians with six or more years of experience more frequently identifying both as factors influencing FDIs compared to those with fewer years of professional experience.

**Table 3:** General knowledge and self-reported practices related to food-drug interactions among registered dietitians by years of experience and area of practice

	Total Population (*n* = 151)	Years of Experience		*p*-valueª	Area of Practice		*p*-valueª
		≤ 5 years (*n* = 62)	≥ 6 years (*n* = 89)		Clinical (*n* = 117)	Non-Clinical (*n* = 34)	
Food and beverages can affect drug therapeutic effects, % (*n*)	99.34 (150)	41.33 (62)	58.67 (88)	0.402	78.00 (117)	22.00 (33)	0.063
Food and beverages can increase toxicity or reduce efficacy, % (*n*)	91.39 (138)	42.03 (58)	57.97 (80)	0.430	76.81 (106)	23.19 (32)	0.520
Age groups at highest risk of FDIs, % (*n*)							
0–17 years	33.11 (50)	44.00 (22)	56.00 (28)	0.605	78.00 (39)	22.00 (11)	0.915
18–45 years	10.60 (16)	37.50 (6)	62.50 (10)	0.760	81.25 (13)	18.75 (3)	0.703
46–64 years	32.45 (49)	44.90 (22)	55.10 (27)	0.506	81.63 (40)	18.37 (9)	0.398
≥ 65 years	86.09 (130)	41.54 (54)	58.46 (76)	0.766	78.46 (102)	21.54 (28)	0.474
Diseases requiring FDI information, % (*n*)							
Cardiovascular	76.16 (115)	40.87 (47)	59.13 (68)	0.932	79.13 (91)	20.87 (24)	0.386
Oncological	79.47 (120)	37.50 (45)	62.50 (75)	0.080	80.83 (97)	19.17 (23)	0.053
Neurodegenerative	61.59 (93)	37.63 (35)	62.37 (58)	0.279	83.87 (78)	16.13 (15)	**0.017**
Endocrine	79.47 (120)	41.67 (50)	58.33 (70)	0.765	78.33 (94)	21.67 (26)	0.623
FDIs involve interactions between the drugs and:, % (*n*)							
Diet (food and beverages)	99.34 (150)	40.67 (61)	59.33 (89)	0.229	77.33 (116)	22.67 (34)	0.589
Food/ Dietary Supplements	33.77 (51)	41.18 (21)	58.82 (30)	0.983	74.51 (38)	25.49 (13)	0.532
Alcohol	28.48 (43)	41.86 (18)	58.14 (25)	0.900	76.74 (33)	23.26 (10)	0.891
Drugs	5.96 (9)	33.33 (3)	66.67 (6)	0.627	44.44 (4)	55.56 (5)	**0.014**
The effects of FDIs are influenced by several factors, such as:, % (*n*)							
Age	49.01 (74)	44.59 (33)	55.41 (41)	0.387	77.03 (57)	22.97 (17)	0.895
Genetic factors	37.75 (57)	52.63 (30)	47.37 (27)	**0.024**	77.19 (44)	22.81 (13)	0.947
Type of diet (enteral/parenteral)	61.59 (93)	39.78 (37)	60.22 (56)	0.687	77.42 (72)	22.58 (21)	0.981
Obesity	39.74 (60)	40.00 (24)	60.00 (36)	0.830	81.67 (49)	18.33 (11)	0.318
Malnutrition	39.74 (60)	41.67 (25)	58.33 (35)	0.902	83.33 (50)	16.67 (10)	0.162
Polypharmacy	84.77 (128)	44.53 (57)	55.47 (71)	**0.041**	75.00 (96)	25.00 (32)	0.085
Drug dosage/ regimen	82.12 (124)	42.74 (53)	57.26 (71)	0.368	76.61 (95)	23.39 (29)	0.583
Diseases (e.g., liver or kidney failure)	74.17 (112)	41.07 (46)	58.93 (66)	0.996	74.11 (83)	25.89 (29)	0.092
Food and beverage intake can interfere with the drug:, % (*n*)							
Absorption	90.67 (136)	40.44 (55)	59.56 (81)	0.861	79.41 (108)	20.59 (28)	0.193
Distribution	78.81 (119)	42.86 (51)	57.14 (68)	0.387	79.83 (95)	20.17 (24)	0.183
Metabolism	48.34 (73)	38.36 (28)	61.64 (45)	0.514	75.34 (55)	24.66 (18)	0.542
Excretion	47.68 (72)	36.11 (26)	63.89 (46)	0.238	81.94 (59)	18.06 (13)	0.210
Drug mechanism of action/ biological target	74.17 (112)	41.07 (46)	58.93 (66)	0.996	78.57 (88)	21.43 (24)	0.588
Routinely ask patients about all medications used, % (n)	94.04 (142)	38.03 (54)	61.97 (88)	**0.003**	78.87 (112)	21.13 (30)	0.104
Most frequently reported FDIs, % (*n=*151)							
Warfarin and vitamin K-rich foods	9.93 (15)	40.00 (6)	60.00 (9)	0.381	80.00 (12)	20.00 (3)	0.498
Grapefruit	6.62 (10)	40.00 (4)	60.00 (6)		80.00 (8)	20.00 (2)	
Iron and dairy products and/or caffeine	5.96 (9)	22.22 (2)	77.78 (7)		66.67 (6)	33.33 (3)	
Levothyroxine and foods	5.30 (8)	25.00 (2)	75.00 (6)		87.50 (7)	12.50 (1)	
Alcohol	4.64 (7)	42.86 (3)	57.14 (4)		85.71 (6)	14.29 (1)	
Teas and antidepressants, contraceptives, antihypertensives, or anticancer drugs	4.64 (7)	57.14 (4)	42.86 (3)		100.00 (7)	0.00 (0)	
Milk and antibiotics	1.99 (3)	0.00 (0)	100.00 (3)		33.33 (1)	66.67 (2)	
Metformin and vitamin B12	1.99 (3)	0.00 (0)	100.00 (3)		100.00 (3)	0.00 (0)	
Vitamin deficiencies associated with PPIs	1.99 (3)	33.33 (1)	66.67 (2)		100.00 (3)	0.00 (0)	
Increase or decrease in the therapeutic effect of the medication	1.99 (3)	0.00 (0)	100.00 (3)		100.00 (3)	0.00 (0)	
Others	4.64 (7)	42.86 (3)	57.14 (4)		71.43 (5)	28.57 (2)	
Did not know/ did not answer	50.33 (76)	48.68 (37)	51.32 (39)		73.68 (56)	26.32 (20)	
Most severe perceived FDIs, % (*n=*151)							
Warfarin and vitamin K-rich foods	12.58 (19)	15.79 (3)	84.21 (16)	0.291	78.95 (15)	21.05 (4)	0.654
Alcohol	8.61 (13)	30.77 (4)	69.23 (9)		84.62 (11)	15.38 (2)	
Grapefruit	5.30 (8)	50.00 (4)	50.00 (4)		50.00 (4)	50.00 (4)	
Anticancer drug-related interactions	2.65 (4)	25.00 (1)	75.00 (3)		75.00 (3)	25.00 (1)	
Increase or decrease in the therapeutic effect of the medication	2.65 (4)	0.00 (0)	100.00 (4)		100.00 (4)	0.00 (0)	
Iron and dairy products and/or caffeine	1.99 (3)	33.33 (1)	66.67 (2)		66.67 (2)	33.33 (1)	
Vitamin deficiencies associated with PPIs	1.32 (2)	50.00 (1)	50.00 (1)		100.00 (2)	0.00 (0)	
Levothyroxine and foods	0.66 (1)	0.00 (0)	100.00 (1)		100.00 (1)	0.00 (0)	
Milk and antibiotics	0.66 (1)	0.00 (0)	100.00 (1)		100.00 (1)	0.00 (0)	
Others	4.64 (7)	42.86 (3)	57.14 (4)		85.71 (6)	14.29 (1)	
Did not know/ Did not answer	58.94 (89)	49.44 (44)	50.56 (45)		76.40 (68)	23.60 (21)	

Data expressed as percentage (*n*)

*Abbreviations*: *FDIs* Food-drug interactions, *PPIs* Proton pump inhibitors

Bold values indicate statistically significant differences (*p *< 0.05)

^a^*p*-values for comparisons between were obtained by the Pearson χ² test

Regarding the conceptual definition of FDIs, interactions may involve not only foods and beverages but also food/ dietary supplements and alcohol. Although nearly all participants identified diet (food and beverages) as relevant (99.34%, *n* = 150), fewer recognised supplementation (33.77%, *n* = 51) and alcohol (28.48%, *n* = 43) as important interaction factors. A significant difference (*p* = 0.014) was observed for selection of the incorrect “drug-drug” option, which was more frequently chosen by registered dietitians outside clinical practice.

Most participants (86.09%, *n* = 130) identified adults aged 65 years or older as the group at highest risk for FDIs. They also prioritised endocrine, oncological, cardiovascular, and neurodegenerative diseases, in this order, as the most critical conditions requiring access to the FDI information. Clinical registered dietitians selected neurodegenerative diseases more frequently than nonclinical registered dietitians (*p* = 0.017).

When asked about mechanisms by which food and beverages may interfere with medications, most respondents (90.67%, *n* = 136) identified absorption as the primary level of interaction, with no significant statistical differences between groups. Regarding clinical practice behaviour, 94.04% (*n* = 142) reported routinely asking patients about all the medications they use. Notably, this practice was more frequent among professionals with more years of experience (*p* = 0.003). For the open-ended question on the most frequent FDIs, more than half of the participants (50.33%, *n* = 76) did not know or did not answer. It should be noted that some of the examples provided by participants do not constitute clearly defined FDIs, and are reported here as stated by respondents without validation. Among those responding, the most cited interaction was warfarin with vitamin K-rich foods (9.93%, *n* = 15), followed by grapefruit-drug interactions (6.62%, *n* = 10) and iron with dairy products and/or caffeine (5.96%, *n* = 9). For perceived most severe FDIs, responses were heterogeneous: 12.58% (*n* = 19) cited warfarin-vitamin K-rich interactions, 8.61% (*n* = 13) alcohol-related interactions, and 5.30% (*n* = 8) grapefruit interactions. Most respondents (58.94%, *n* = 89) did not provide an answer. Although no significant differences were observed between subgroups for these open-ended questions, responses were predominantly provided by registered dietitians with six or more years of experience and from those in clinical practice (Table 3). 

Regarding specific FDI knowledge (Table 4), important gaps were observed for clinically relevant interactions involving propranolol and warfarin. Only 22.52% (*n* = 34) correctly identified that a protein-rich diet should be avoided with propranolol. For warfarin-related interactions, 42.38% (*n* = 64) of participants recognised that garlic and ginger may increase bleeding risk, and 62.25% (*n* = 94) correctly identified that consuming spinach, broccoli, and cauliflower may reduce anticoagulant efficacy and increase thrombotic risk. For theophylline interactions, 25.17% (*n* = 38) were unaware that asthmatic patients treated with this medicine should avoid coffee, tea, and chocolate. Clinical registered dietitians demonstrated greater knowledge of these FDIs, with a statistically significant difference (*p* = 0.041). Regarding tetracyclines and fluoroquinolones, 31.13% (*n* = 47) of respondents were unaware that milk and dairy products should be avoided. Registered dietitians with six or more years of experience showed significantly greater knowledge (*p* = 0.017). For the remaining FDI questions, 69% to 74% of participants answered correctly, with no significant subgroup differences.

**Table 4:** Specific food-drug interaction knowledge among registered dietitians by years of experience and area of practice

	Total Population (*n* = 151)	Years of Experience		*p*-valueª	Area of Practice		*p*-valueª
		≤ 5 years (*n* = 62)	≥ 6 years (*n* = 89)		Clinical (*n* = 117)	Non-Clinical (*n* = 34)	
Specific FDIs, % (*n*)							
Theophylline: avoid coffee, tea, and chocolate	74.83 (113)	42.48 (48)	57.52 (65)	0.541	73.45 (83)	26.55 (30)	**0.041**
Tetracyclines/fluoroquinolones: avoid milk and dairy products	68.87 (104)	34.62 (36)	65.38 (68)	**0.017**	77.88 (81)	22.12 (23)	0.861
MAOIs: avoid cheese, wine, and fermented foods	68.87 (104)	36.54 (38)	63.46 (66)	0.093	74.04 (77)	25.96 (27)	0.132
Warfarin: vitamin K–rich vegetables reduce efficacy (thrombotic risk)	62.25 (94)	41.49 (39)	58.51 (55)	0.890	74.47 (70)	25.53 (24)	0.255
Warfarin: garlic and ginger increase efficacy (bleeding risk)	42.38 (64)	43.75 (28)	56.25 (36)	0.564	78.13 (50)	21.88 (14)	0.871
Nifedipine/simvastatin: avoid grapefruit juice	71.52 (108)	40.74 (44)	59.26 (64)	0.900	76.85 (83)	23.15 (25)	0.768
Propranolol: avoid a protein-rich diet	22.52 (34)	32.35 (11)	67.65 (23)	0.241	73.53 (25)	26.47 (9)	0.531
St. John’s wort: reduces efficacy of immunosuppressants/ oral contraceptives	74.17 (112)	38.39 (43)	61.61 (69)	0.259	76.79 (86)	23.21 (26)	0.728
Drug-alcohol interactions, % (*n*)							
Metronidazole/cefoperazone: avoid alcohol	65.56 (99)	42.42 (42)	57.58 (57)	0.628	79.80 (79)	20.20 (20)	0.637
Alcohol does not affect all antibiotics	43.71 (66)	39.39 (26)	60.61 (40)	0.698	80.30 (53)	19.70 (13)	0.765
Acute alcohol: decreases metabolism (increases drug effects)	21.85 (33)	33.33 (11)	66.67 (22)	0.486	75.76 (25)	24.24 (8)	0.964
Chronic alcohol: increases metabolism (reduces drug effects)	88.08 (133)	40.60 (54)	59.40 (79)	0.380	78.20 (104)	21.80 (29)	0.116

Data expressed as percentage (*n*)

*Abbreviations*: *FDIs* Food-drug interactions, *MAOIs* Monoamine oxidase inhibitors

Bold values indicate statistically significant differences (*p *< 0.05)

^a^*p*-values for comparisons were obtained using the Pearson χ² test, as appropriate

On drug-alcohol interactions, most participants (88.08%, *n* = 133) correctly identified that chronic alcohol consumption can increase drug metabolism and reduce the efficacy of several medications. However, only 33 participants (21.85%) recognised that acute or occasional alcohol intake may reduce metabolism and potentially increase drug effects. Less than half of the participants (43.71%, *n* = 66) correctly recognised that alcohol consumption does not interact with all antibiotics. However, 65.56% (*n* = 99) correctly identified the need to avoid alcohol with specific antibiotics, such as metronidazole and cefoperazone. No significant subgroup differences were observed for alcohol-related items. Finally, the mean specific FDI knowledge score for clinical registered dietitians was 76.60%, calculated as the mean of correct response percentages across all items in Table 4 for this subgroup (data not shown). 

**Fig. 1:**
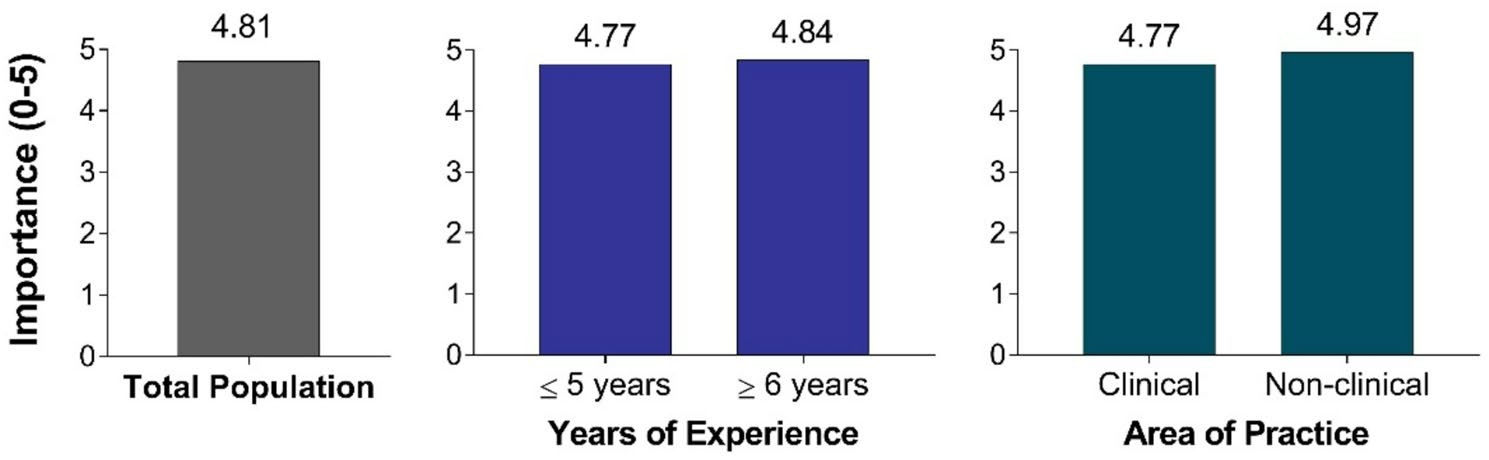
Importance attributed by registered dietitians to patient counselling on food-drug interactions (scale 0–5)

Lastly, the questionnaire explored registered dietitians’ perceptions regarding the importance and dissemination of FDI knowledge. Most participants considered patient education on FDIs essential, with a high mean importance rating of 4.81 ± 0.45, (scale 0–5), as shown in Fig. 1. Although minor variations in ratings across groups were observed, no statistically significant differences were found between experience and practice groups.

**Fig. 2:**
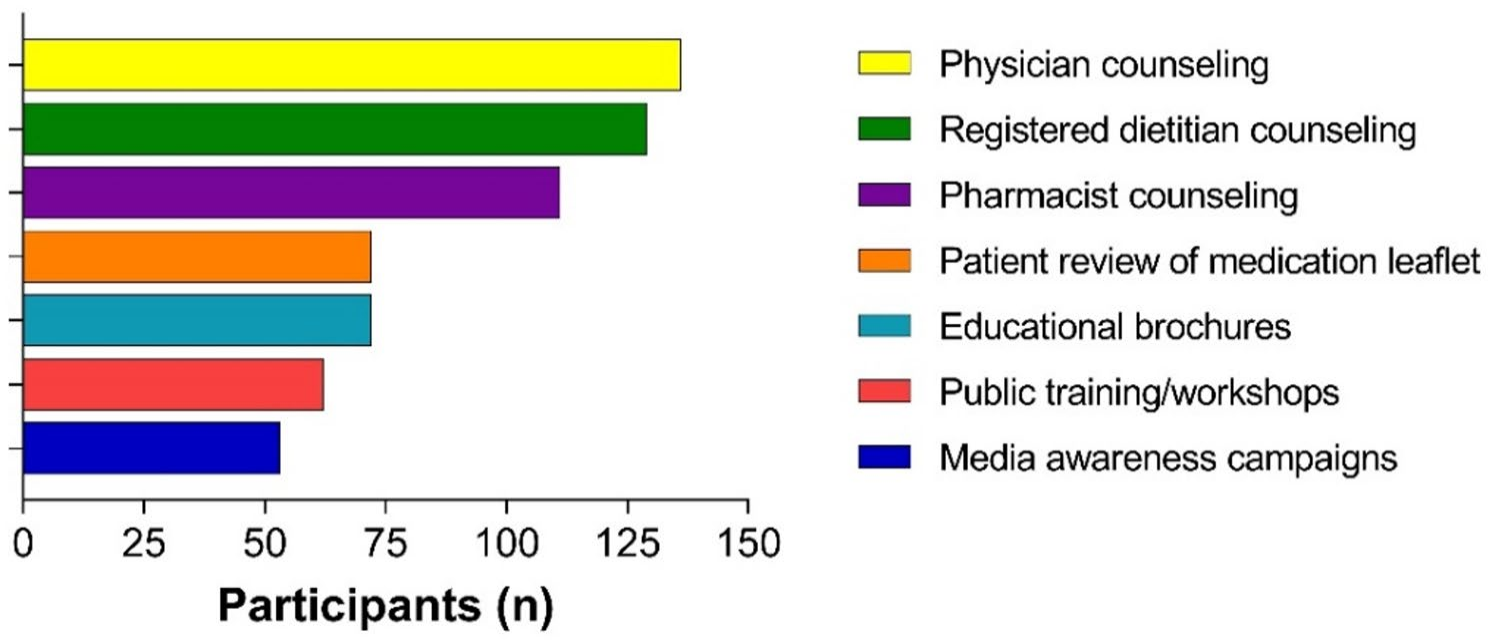
Perceived effective strategies to increase public awareness of food-drug interactions among registered dietitians

Registered dietitians identified the most effective strategies to increase public awareness of FDIs as explanations provided by physicians during prescribing (*n* = 136), counselling provided by registered dietitians during consultations (*n* = 129), and information from pharmacists at dispensing (*n* = 111) (Fig. 2). No significant differences were observed between subgroups.

Finally, when asked about the importance of staying updated on FDIs to provide accurate patient guidance, almost all participants (98.68%, *n* = 149) responded affirmatively, with no statistically significant differences across subgroups (data not shown).

## Discussion

The present study provides novel evidence on the knowledge and competence of registered dietitians in FDIs, extending previous research on nutrition students [31] to the practising nutrition workforce. This study is pioneering in assessing the knowledge of registered dietitian members of the Order of Nutritionists (*Ordem dos Nutricionistas*) regarding both general concepts and specific examples of FDIs.

When analysing the participants’ academic backgrounds, it was found that registered dietitians who did not receive training on FDIs had six or more years of experience. This finding suggests that the FDI content has been more consistently incorporated into nutrition curricula in recent years, reflecting evolving recognition of medication-nutrition safety within nutrition sciences education.

A closer examination of general knowledge about FDIs revealed that while most participants could answer basic questions, they struggled with more complex ones. This pattern is consistent with educational models in which foundational concepts are acquired during undergraduate training, whereas clinically nuanced FDI knowledge requires applied learning and practice exposure. This difficulty likely stems from insufficient training in FDIs, highlighting a gap in registered dietitians’ deeper understanding.

When asked about the age group most at risk for FDIs, it was expected that the majority of participants would select the option “Over 64 years old,” given the higher incidence of polypharmacy among older adults [33]. According to Machado-Alba et al. [34], the prevalence of polypharmacy in the elderly ranges from 30% to 70%, and can even reach up to 90% among those institutionalised in residential or healthcare facilities. Furthermore, older people are more likely to develop renal insufficiency [35] and liver insufficiency [36], which significantly affect drug pharmacokinetics and pharmacodynamics, thereby increasing the risk of FDIs [37, 38]. However, only 65 registered dietitians selected this option exclusively, representing just 43.05% of the sample who correctly answered this question. Regarding the prioritisation of disease groups requiring FDI awareness, participants identified endocrine, oncological, cardiovascular, and neurodegenerative conditions as the most critical, in this order. This prioritisation is clinically meaningful, as these conditions are associated with some of the most extensively documented FDIs in the literature [39]. Endocrine conditions involve well-documented dietary interactions with levothyroxine and antidiabetic agents; oncological conditions carry a high FDI burden given the narrow therapeutic index of many chemotherapy agents; cardiovascular diseases encompass interactions such as warfarin–vitamin K and grapefruit with statins or calcium channel blockers; and neurodegenerative conditions include drugs such as levodopa with high-protein diets [39]. The prioritisation of these four disease groups therefore reflects a degree of applied clinical awareness among participants, even in the context of broader knowledge gaps, particularly among older adults with multimorbidity and polypharmacy.

When analysing the number of correct responses to the concept of “Drug-Food Interaction”, a notable knowledge gap emerged. Most of the registered dietitians surveyed believed that the concept refers solely to the interaction between one or more drugs and the diet (food and beverages), as the term suggests. However, only 32 registered dietitians, approximately one-fifth of the sample, provided the correct answer, which also includes food/dietary supplements and alcohol in addition to diet. Misclassification of supplements and alcohol as outside the FDIs framework may have practical implications, given the growing prevalence of food supplements use in populations [40].

According to the National Institutes of Health (NIH), the U.S. Biomedical and Public Health Research Agency, a “Drug-Food Interaction” is defined as a reaction between a drug (or drugs) and food, beverages (including alcoholic beverages), or dietary supplements [41]. This explanation highlights a gap in health literacy, even among healthcare professionals, particularly regarding dietary supplements. These supplements are food products, but are often mistakenly associated with medications [42].

The consumption of food or dietary supplements has been steadily rising each year [40, 43], which increases the likelihood that nutrition professionals will encounter patients who use these products. Beyond their pharmacological interactions with medications [44], food supplements may also pose additional risks due to contamination and adulteration [45], further underscoring the critical role of registered dietitians in assessing supplement use during dietary consultations [46]. These findings reinforce the need for registered dietitians to integrate supplement-drug considerations into routine dietetic assessment and counselling.

As the analysis of questionnaire responses continued, particularly in the section on specific knowledge of FDIs, a noticeable disparity emerged. While some questions yielded relatively high percentages of correct answers (~ 75%), others yielded significantly lower accuracy rates (~ 20%). This discrepancy underscores the variation in participants’ knowledge across the different questions. Such variability suggests that exposure to FDIs in practice may be drug-specific rather than conceptually comprehensive.

Overall, approximately three-quarters of the registered dietitians surveyed were knowledgeable about the foods to avoid when administering certain drugs or therapeutic groups, such as theophylline, tetracyclines, monoamine oxidase inhibitors (MAOIs), nifedipine, and immunosuppressants. This finding aligns with a previous study by Radwan et al. (2018), which also highlighted that despite the generally limited knowledge of FDIs among healthcare professionals, there was a higher percentage of correct answers for specific drugs like tetracyclines (94.21%), theophylline (74.13%), and MAOIs (71.81%).

Higher recognition of interactions involving frequently discussed or clinically emphasised drugs likely reflects experiential learning during patient care and professional practice. As more patients require treatments for conditions such as psychiatric and respiratory diseases, registered dietitians are compelled to deepen their knowledge about the related medicines. Additionally, the need to address acute conditions or those with limited therapeutic success, such as infections, likely heightens awareness among both registered dietitians and patients about the potential interactions between certain foods and these medications.

Contrary to expectations, knowledge accuracy declined notably for interactions involving propranolol and warfarin. Propranolol showed the lowest correct response rate in the study (22.52%), suggesting limited awareness of its interaction with high-protein diets, a finding with potential clinical relevance in nutritional counselling. Regarding warfarin, while recognition of vitamin K-rich food interactions was moderate (62.25%), familiarity with the potentiating effect of garlic and ginger on bleeding risk was considerably lower (42.38%). This is particularly concerning given warfarin’s narrow therapeutic window and its sensitivity to dietary factors [47]. Together, these findings suggest that knowledge gaps are most pronounced for FDIs requiring nuanced dietary management, with direct implications for patient safety in dietetics practice. Additionally, the high non-response rate to open-ended items, with more than half of participants (50.33%) unable to name the most frequent FDI encountered in practice, and 58.94% unable to identify the most severe, is itself a clinically significant finding. This pattern likely reflects genuine uncertainty in the applied recall and recognition of FDIs beyond what closed-format knowledge items capture, and underscores that declarative knowledge alone may not translate to clinical readiness. Furthermore, the fact that a proportion of participants provided responses that do not constitute clearly defined FDIs, such as general references to therapeutic effects, suggests that knowledge gaps extend beyond recall to a conceptual level, reflecting uncertainty about what constitutes a FDI *per se*.

To facilitate comparison with other studies, the specific knowledge of registered dietitians regarding FDIs was categorised into five levels: Insufficient (< 50%), Sufficient (50%-64%), Good (65%-79%), Very Good (80%-89%), and Excellent (90%-100%). The average percentage of correct answers among the 151 registered dietitians in this study was 58.72%, indicating their knowledge was “Sufficient”, though near the lower end of the range. This result shows a significant opportunity to improve registered dietitians’ understanding of FDIs. However, compared to existing scientific evidence, this study’s findings on specific knowledge about FDIs are relatively higher. Previous research involving healthcare professionals, such as doctors, nurses, pharmacists, and registered dietitians, has consistently shown inadequate, low, or insufficient knowledge about FDIs, revealing significant gaps, particularly in identifying foods that interact with specific drugs [6, 25, 26, 28–30, 32, 48, 49].

It is important to note that when comparing the average percentage of correct answers across groups based on registered dietitians’ area of practice and years of experience, the group with 6 or more years of professional experience had a slightly higher average. Despite this improvement, the average remains in the “Sufficient” category, with no significant difference from the overall group. This finding contrasts with the study performed by Benni et al. (2012), which reported a positive correlation between years of professional experience and FDI knowledge among healthcare professionals [32].

Conversely, clinical registered dietitians achieved an average score of 76.60%, placing them in the “Good” knowledge category, compared to the “Sufficient” category of the overall sample. This result supports the hypothesis that routine exposure to medication-nutrition issues in clinical settings enhances applied FDI competence. This outcome was expected, as clinical registered dietitians regularly engage with patients in hospital or clinical settings, frequently addressing FDIs in their daily practice.

Given that registered dietitians play a crucial role in public health, a comprehensive understanding of FDIs should ideally be widespread across all practice areas. However, this was not observed among more experienced registered dietitians, indicating that direct patient care, practical exposure, and clinical engagement may be more influential than years in the profession alone. In addition to dietitians working in general clinical practice, professionals involved in specialised fields requiring close coordination between nutrition and pharmacological treatments, such as oncology nutrition, may also develop greater awareness of FDIs through routine contact with complex therapeutic regimens. Importantly, few previous studies have stratified FDI knowledge among registered dietitians by practice area, limiting direct comparison and underscoring the contribution of the present findings.

Notably, these findings are consistent with our previous study in nutrition students, which demonstrated foundational awareness but limited depth in clinically relevant FDI knowledge [31]. Together, the student and professional data suggest that gaps identified during training may persist into practice unless reinforced through continuing professional education. To address this issue, there is a pressing need for training organisations to develop and offer more comprehensive courses on FDIs. This opinion is echoed by other researchers in recent years, who emphasise the importance of ongoing education and continuous updates on FDIs to ensure that healthcare professionals remain well-informed [26, 30, 32, 48].

A compelling example of the necessity of targeted training is the study by Abbasi Nazari et al., which demonstrated that implementing training programmes on FDIs in a hospital setting reduced interactions by approximately 10% [50]. The study showed a notable decline in FDIs during critical phases, such as drug absorption, following nurses’ training sessions. These data support the potential effectiveness of structured FDI education in improving medication-nutrition safety across healthcare professions, including registered dietitians.

### Strengths and limitations

A major strength of the present study is its focus on registered dietitians at the national level, providing a pioneering, comprehensive assessment of FDI knowledge within the nutrition workforce. By including participants from across mainland Portugal and the autonomous regions and representing graduates from most Portuguese institutions offering nutrition degrees, the sample captured diverse educational and professional backgrounds. This broad representation enhances the relevance of the findings to contemporary dietetics practice in Portugal. Additionally, the inclusion of registered dietitians from multiple areas and with varying levels of professional experience enabled stratified analyses of FDI knowledge by clinical exposure and career stage. This diversity allows for more realistic, detailed, and comparative perceptions based on these variables. Such stratification is rarely reported in previous FDI studies and contributes to a more nuanced understanding of competence patterns within the profession. Another strength is the combined evaluation of educational exposure and applied FDI knowledge, enabling exploration of potential associations between training and competence. This approach provides evidence relevant to curriculum development and continuing professional education for registered dietitians.

Nevertheless, some limitations should be considered. No formal sample size calculation was performed, as participants were recruited through convenience sampling, which may affect the precision of estimates and the generalisability of findings. Data were collected through a self-reported online questionnaire without time constraints. This may have allowed participants to consult external sources during completion, potentially overestimating actual clinical knowledge and recall. Future studies should consider timed knowledge assessments to better approximate real-world clinical competence. Self-report measures may also be influenced by social desirability and recall bias, particularly regarding prior educational exposure. Participation was voluntary, and respondents may have been more interested in FDIs, particularly those working in clinical nutrition. It also should be noted that the five-level classification used to categorise knowledge scores (Insufficient < 50%; Sufficient 50–64%; Good 65–79%; Very Good 80–89%; Excellent 90–100%) was adapted by the authors from percentage-based grading conventions and does not derive from a validated instrument. The arbitrary nature of these thresholds limits the comparability of findings and should be considered when interpreting results. Additionally, the results section reports multiple statistical comparisons across knowledge items, stratified by years of experience and area of practice, without correction for multiple testing. Given the exploratory nature of this study, formal correction was not applied; however, findings with p-values near the significance threshold (e.g., genetic factors or neurodegenerative diseases) should be interpreted with caution. Finally, as this was a cross-sectional study with data collected at a single time point, causal relationships between training exposure and FDI knowledge cannot be inferred. Future studies with larger and more diverse samples and longitudinal designs are warranted to confirm and extend these findings.

## Conclusion

Registered dietitians demonstrated gaps in clinically relevant FDI knowledge, particularly for specific and high-risk interactions, indicating opportunities to strengthen medication-nutrition competence in dietetics practice. Clinical registered dietitians and those with more years of professional experience generally demonstrated greater knowledge of FDIs and were more likely to pursue additional training in this area.

These findings underscore the need to enhance both foundational and applied FDI education across nutrition training and continuing professional development. Strengthening FDI competence is essential to support accurate dietary guidance, optimise therapeutic outcomes, and promote patient safety among populations exposed to polypharmacy.

Targeted educational strategies and practice-oriented training may help ensure that registered dietitians across all practice areas are adequately prepared to integrate medication considerations into nutrition care.

## Data Availability

The data collection instrument used in this study is not publicly available but may be provided upon reasonable request to the corresponding author.
